# Polycarbonate Bottle Use and Urinary Bisphenol A Concentrations

**DOI:** 10.1289/ehp.0900604

**Published:** 2009-05-12

**Authors:** Jenny L. Carwile, Henry T. Luu, Laura S. Bassett, Daniel A. Driscoll, Caterina Yuan, Jennifer Y. Chang, Xiaoyun Ye, Antonia M. Calafat, Karin B. Michels

**Affiliations:** 1 Department of Epidemiology, Harvard School of Public Health and; 2 Harvard College, Faculty of Arts and Sciences, Harvard University, Cambridge, Massachusetts, USA; 3 Division of Laboratory Sciences, National Center for Environmental Health, Centers for Disease Control and Prevention, Atlanta, Georgia, USA; 4 Obstetrics and Gynecology Epidemiology Center, Department of Obstetrics, Gynecology and Reproductive Biology, Brigham and Women’s Hospital, Harvard Medical School, Boston, Massachusetts USA

**Keywords:** biomarkers, bisphenol A, endocrine disruptors, human, polycarbonate plastic

## Abstract

**Background:**

Bisphenol A (BPA) is a high-production-volume chemical commonly used in the manufacture of polycarbonate plastic. Low-level concentrations of BPA in animals and possibly in humans may cause endocrine disruption. Whether ingestion of food or beverages from polycarbonate containers increases BPA concentrations in humans has not been studied.

**Objectives:**

We examined the association between use of polycarbonate beverage containers and urinary BPA concentrations in humans.

**Methods:**

We conducted a nonrandomized intervention of 77 Harvard College students to compare urinary BPA concentrations collected after a washout phase of 1 week to those taken after an intervention week during which most cold beverages were consumed from polycarbonate drinking bottles. Paired *t*-tests were used to assess the difference in urinary BPA concentrations before and after polycarbonate bottle use.

**Results:**

The geometric mean urinary BPA concentration at the end of the washout phase was 1.2 μg/g creatinine, increasing to 2.0 μg/g creatinine after 1 week of polycarbonate bottle use. Urinary BPA concentrations increased by 69% after use of polycarbonate bottles (*p* < 0.0001). The association was stronger among participants who reported ≥ 90% compliance (77% increase; *p* < 0.0001) than among those reporting < 90% compliance (55% increase; *p =* 0.03), but this difference was not statistically significant (*p =* 0.54).

**Conclusions:**

One week of polycarbonate bottle use increased urinary BPA concentrations by two-thirds. Regular consumption of cold beverages from polycarbonate bottles is associated with a substantial increase in urinary BPA concentrations irrespective of exposure to BPA from other sources.

The endocrine-disrupting chemical bisphenol A (BPA) has recently garnered heightened attention because of widespread human exposure and disruption of normal reproductive development in laboratory animals [Center for the Evaluation of Risks to Human Reproduction (CERHR) 2008; Chapin et al. 2008; Goodman et al. 2006; European Union 2003; vom Saal and Hughes 2005]. BPA is thought to disrupt normal cell function by acting as an estrogen agonist (Wozniak et al. 2005) as well as an androgen antagonist (Lee et al. 2003). In animal studies, prenatal and neonatal exposure to BPA has been linked to early onset of sexual maturation (Howdeshell et al. 1999), altered development and tissue organization of the mammary gland (Markey et al. 2001), induction of pre neoplastic mammary gland (Durando et al. 2007) and reproductive tract lesions (Newbold et al. 2007), increased prostate size (Timms et al. 2005), and decreased sperm production (vom Saal et al. 1998) in offspring. Most recently, exposure to BPA has also been associated with chronic disease in humans, including cardiovascular disease, diabetes, and serum markers of liver disease (Lang et al. 2008).

Orally administered BPA is rapidly metabolized by glucuronidation during first-pass metabolism, with a biological half-life of approximately 6 hr and nearly complete elimination within 24 hr (Volkel et al. 2002). However, because of continuous and widespread exposure, > 92% of the 2,517 participants ≥ 6 years of age in the U.S. 2003–2004 National Health and Nutrition Examination Survey (NHANES) had detectable concentrations of BPA in their urine (Calafat et al. 2008). The geometric mean (GM) urinary BPA concentration in that study was 2.6 μg/L (2.6 μg/g creatinine), and the 95th percentile was 15.9 μg/L (11.2 μg/g creatinine).

An important source of human exposure is thought to be the ingestion of food and drink that has been in contact with epoxy resins or polycarbonate plastics (Kang et al. 2006). Polycarbonate is a durable, lightweight, and heat-resistant plastic, making it popular for use in plastic food and beverage containers. Indeed, nearly three-fourths of the 1.9 billion pounds of BPA used in the United States in 2003 was used for the manufacture of poly-carbonate resin (CERHR 2008). Other common uses of BPA include the manufacture of epoxy resins used as composites and sealants in dentistry and in the lacquer lining of aluminum food and beverage cans (CERHR 2008; European Union 2003).

Laboratory studies have demonstrated that biologically active BPA is released from polycarbonate bottles after simulated normal use (Brede et al. 2003; Le et al. 2008). High temperatures as well as acidic and alkali solutions cause polymer degradation via hydrolysis, resulting in increased BPA migration. After incubation for 8, 72, and 240 hr in food-simulating solvents (10% ethanol at 70°C and corn oil at 100°C), mean BPA migration increased with incubation time (Onn Wong et al. 2005). After a sequence of washing and rinsing, Le et al. (2008) found that new poly-carbonate bottles leached 1.0 ± 0.3 μg/mL BPA (mean ± SD) into the bottle content after incubation at room temperature for 7 days. Although exposure to boiling water increased the rate of BPA migration up to 55-fold, used bottles did not leach significantly more BPA than new ones. However, other studies have found that higher concentrations of BPA leach from used polycarbonate plastic than from new. BPA has been observed to leach from polycarbonate animal cages after 1 week of incubation at room temperature, with higher levels of migration from used versus new cages (Howdeshell et al. 2003). Similarly, after incubation in 100°C water for 1 hr, the amount of BPA leached from baby bottles subjected to simulated use (including dishwashing, boiling, and brushing into the bottle) exceeded the amount that leached from new baby bottles (Brede et al. 2003).

Recently, some polycarbonate bottle manufacturers voluntarily eliminated BPA from their products (Nalgene Outdoor 2008), and several retailers withdrew polycarbonate bottles from their stores altogether (Mui 2008). Canada has imposed a ban on the use of BPA in poly-carbonate baby bottles in order to reduce exposure of infants to BPA (Health Canada 2008), and similar legislation is being considered by several U.S. states (Austen 2008). However, such actions have been largely preemptive, as no epidemiologic study has evaluated the physiologic consequences of polycarbonate bottle use. Therefore, we studied the impact of cold beverage consumption from poly-carbonate bottles on measurable urinary BPA concentrations among a Harvard College population. We also measured exposure to the phenols triclosan (TCS), methyl paraben (MePB), propyl paraben (PrPB), and benzo-phenone-3 (BP-3), which occurs mainly through the use of personal care products. Therefore, because exposure of these chemicals is considered unrelated to polycarbonate bottle use, we assessed their association with poly-carbonate bottle use as a negative control.

## Materials and Methods

### Study population

We recruited Harvard College students in April 2008 via e-mails to freshman dormitory, upperclass house, and student organization mailing lists. Students were directed to a survey website, where they provided contact information and indicated their availability for the study dates. Participant instructions and informed consent forms were also made available. Students at least 18 years of age who were available for the entire study period were considered eligible and were invited to an introductory meeting. The 89 students who attended the meeting returned their signed informed consent forms, provided demographic information (age, sex, ethnicity), and received two stainless steel bottles. Seven participants withdrew from the study before completing the washout phase, and five participants withdrew after completing the washout phase but before completion of the intervention phase. Participants who withdrew were similar to those who completed the study in terms of age (median, 19 years; range, 18–22 years) but were slightly more likely to be female (66.7%) than students who completed the entire study. A total of 77 participants completed the study. A $25 compensation was provided only upon completion of the study. The study was approved by the Human Studies Institutional Review Board of Harvard University. The involvement of the Centers for Disease Control and Prevention (CDC) laboratory was limited and was determined not to constitute engagement in human subjects research.

### Study design

The study began with a 7-day washout phase designed to minimize exposure to BPA by limiting the consumption of cold beverages to those contained in stainless steel bottles. Because orally administered BPA is rapidly excreted (Volkel et al. 2002), we considered a 1-week washout period sufficient. We provided participants with two stainless steel bottles and advised them to drink all cold beverages from the stainless steel bottles and avoid drinking water from #7 polycarbonate plastic cold water dispensers available in college dining halls. Participants donated urine on their choice of 2 of 3 final days of the wash-out phase. Urine donation took place between 1700 and 2000 hours on two of the days, and between 1600 and 1900 hours on the third day. Two polycarbonate bottles were distributed to each participant on the second day of urine donation during the washout phase. Participants were advised to begin drinking all cold beverages from the polycarbonate bottles (intervention week) immediately. Urine was donated again on the participant’s choice of 2 of 3 final days of the week of polycarbonate bottle use between 1700 and 2000 hours. On the final day of urine donation, participants completed a brief questionnaire in which they estimated their percentage compliance during the week in which they were asked to drink cold beverages from the polycarbonate bottles.

Stainless steel bottles (27 fluid ounces, with #5 polypropylene loop cap) were obtained from Kleen Kanteen (763332017107; Chico, CA). Polycarbonate bottles [Nalgene 32 fluid ounce, Lexan narrow mouth (#53175), and Lexan wide mouth (#53107)] were obtained from Karst Sports (Shinnston, WV). All participants were permitted to keep the bottles used in the study.

### Urine sample collection

Urine was collected in a polypropylene container, aliquoted, and frozen at −20°C. After study completion, samples were defrosted at 4°C overnight and vortexed; equal volumes of the two samples from each phase of the study were then combined and aliquoted. We shipped aliquots of samples (blinded to those performing laboratory analyses) on dry ice overnight to the CDC for measuring BPA and other urinary phenol concentrations; samples were also taken to N. Rifai (Children’s Hospital, Boston, MA) for analysis of urinary creatinine.

### Urinary phenol concentrations

Total urinary concentrations (free plus conjugated species) of BPA and the other four phenols were determined using online solid-phase extraction coupled to isotope dilution high-performance liquid chromatography (HPLC)-tandem mass spectrometry (MS/MS) on a system constructed from several HPLC Agilent 1100 modules (Agilent Technologies, Wilmington, DE) coupled to a triple quadrupole API 4000 mass spectrometer (Applied Biosystems, Foster City, CA) (Ye et al. 2005). First, 100 μL urine was treated with β-glucuronidase/sul-fatase (*Helix pomatia*; Sigma Chemical Co., St. Louis, MO) to hydrolyze conjugated species of the phenols. The phenols were then retained and concentrated on a C18 reversed-phase size-exclusion solid-phase extraction column (Merck KGaA, Darmstadt, Germany), separated from other urine matrix components using a pair of monolithic HPLC columns (Merck KGaA), and detected by nega tive ion-atmospheric pressure chemical ionization-MS/ MS. The limits of detection (LODs) in a 0.1-mL urine sample were 0.4 μg/L (BPA and BP-3), 0.2 μg/L (PrPB), 1.0 μg/L (MePB), and 2.3 μg/L (TCS). Low-concentration (~ 4 to ~ 25 μg/L) and high-concentration (~ 10 to ~ 65 μg/L) quality-control materials, prepared with pooled human urine, were analyzed with standard, reagent blank, and unknown samples (Ye et al. 2005). Creatinine was measured by a rate-blanked method using the Hitachi 917 analyzer and Roche Diagnostics reagents (both from Roche Diagnostics, Indianapolis, IN).

### Statistical analysis

Urinary phenol concentrations were normalized for dilution using the formula 100 × urinary phenol concentration (micrograms per liter) ÷ creatinine concentration (milli grams per deciliter). Creatinine-adjusted phenol concentrations (micrograms per gram creatinine) were not normally distributed and were therefore log-transformed. Phenol concentrations < LOD were assigned a value equal to one-half the LOD (Hornung 1990) prior to creatinine adjustment.

We calculated GMs for creatinine- corrected concentrations. We used paired *t*-tests to examine the association between log-transformed urinary creatinine-adjusted phenol concentrations and drinking-container assignment overall and within subsets defined by percent compliance during the intervention phase (≥ median and < the median). When the participant reported compliance as a range, we used the mean. Two sample *t*-tests were used to make comparisons between the strata defined by percent compliance.

## Results

The study population included 77 subjects who ranged in age from 18 to 23 years, with a median of 19 years (Table 1). On the basis of self-reported data, we categorized race/ ethnicity into four groups: Caucasian, Asian, African American, and Hispanic. Thirty participants (39.0%) were Caucasian, 38 were of Asian descent (49.4%), 5 were African American (6.5%), and 4 were Hispanic (5.2%). Forty-one subjects were male (53.3%). Protocol compliance for the week in which participants drank from polycarbonate bottles ranged from 50% to 100% but was generally high, with a median of 90%.

Nine samples (11.7%) from the washout week and three samples (3.9%) from the intervention week (period in which participants drank from polycarbonate bottles) had BPA concentrations < LOD. BP-3 and MePB were detected in all participants, and PrPB was detected in all but one participant each week. TCS was detected in 75.3% of the samples taken at the end of the washout week and in 74.0% of the samples collected after the intervention week. The GM concentration of BPA was 1.3 μg/L (1.2 μg/g creatinine) during the washout period and 2.1 μg/L (2.0 μg/g creatinine) during the intervention week (Table 2). GM concentrations for the washout phase and intervention week were 46.1 and 66.8 μg/g creatinine for BP-3; 51.3 and 48.4 μg/g creatinine for MePB; 8.4 and 8.8 μg/g creatinine for PrPB; and 15.5 and 17.3 μg/g creatinine for TCS, respectively.

Table 3 presents results from paired *t*-tests comparing urinary BPA concentrations in weeks 1 and 2. Urinary BPA concentrations increased by 69% after polycarbonate bottle use. We observed a larger difference between the intervention and washout weeks in the stratum with intervention compliance ≥ 90% (77% increase; *p <* 0.0001) relative to the stratum with compliance < 90% (55%; *p =* 0.03); however, the strata were not significantly different from each other (*p =* 0.54). Of the other phenols, only urinary BP-3 concentration was associated with polycarbonate bottle use, with relatively higher concentrations observed after use of polycarbonate bottles (45% increase; *p =* 0.001). A slightly larger change in BP-3 concentration was observed in the less compliant stratum (64% increase; *p =* 0.01) relative to the more compliant stratum (36% increase; *p =* 0.04); however, this difference was not statistically significant (*p =*0.42).

## Discussion

Several previous studies have demonstrated that biologically active BPA is released from polycarbonate bottles into the bottle content after simulated normal use (Brede et al. 2003; Le et al. 2008). However, to our knowledge, the present study is the first to quantify the corresponding increase in urinary BPA concentrations after use of polycarbonate drinking bottles. Thus, this study suggests that BPA-containing drinking vessels release sufficient amounts of BPA into the bottle content to significantly raise the amount of BPA excreted in urine in humans who drink from these bottles. Specifically, in this study of 77 Harvard College students, urinary BPA concentrations were higher when participants consumed the majority of cold beverages from polycarbonate bottles compared with a washout phase in which polycarbonate bottles were avoided. This statistically significant increase was observed despite background BPA exposure from other sources, which was not assessed nor controlled in this study. This association persisted after stratification by self-reported compliance during the intervention week, with a nonsignificantly larger difference between intervention and washout phase urinary BPA concentrations among participants reporting higher percent compliance. Of interest, the urinary BPA concentrations reported for this group of students (both before and after the intervention) were similar to those reported for the U.S. general population (Calafat et al. 2008) and selected populations in Southeast Asia (Kim et al. 2003; Matsumoto et al. 2003; Ouchi and Watanabe 2002; Yang et al. 2003).

Because of BPA’s short half-life and rapid elimination (Volkel et al. 2002), carryover of ingested BPA between the washout phase and intervention phase was considered unlikely. It is possible that certain subject characteristics may have varied between the 2 weeks, producing a period effect that was unaccounted for by our analyses. We considered this improbable because of the lack of variability in the routine of undergraduate students, who attended the same classes and ate in the same campus dining halls each week. Additionally, the similarity of observed urinary BPA concentrations to national levels suggests that subjects were exposed to typical amounts of BPA from other sources during both weeks. Moreover, fatigue and the participants’ exposure to mass media concerning the leaching of BPA from poly-carbonate bottles might have induced better compliance during the washout phase than the intervention phase, thus leading to an underestimate of the impact of polycarbonate bottle use on urinary BPA concentrations. It is also possible that participants may have modified their behavior during the week of polycarbonate bottle use to reduce BPA exposure from other sources. However, other sources of BPA exposure have not been well publicized, and any reduction in exposure to other sources of BPA during the intervention week would have reduced the observed effect estimate.

We used spot urine samples for convenience; however, disadvantages of this method include interperson variability in BPA concentration and variability in the volume of urine (Barr et al. 2005). Two equal-volume samples from each week were combined to minimize day-to-day variability. Additionally, we collected all urine samples in the evening, minimizing variability related to time of day (Mahalingaiah et al. 2008). Concern regarding interperson variability is also mitigated by recent findings that a single urinary BPA concentration was predictive of long-term exposure on a scale of weeks to months (Mahalingaiah et al. 2008). Urinary BPA concentrations were creatinine-adjusted to account for variability in urine dilution. Overall, the results obtained after the analysis with and without correction of the urinary dilution were fairly similar. However, failure to control for urinary creatinine concentrations resulted in a greater degree of within-person variation and, subsequently, decreased precision, as evidenced by wider 95% CIs. For this reason, we have presented only the creatinine-adjusted results.

To account for the possibility of a chance finding, we also compared the impact of poly-carbonate bottle use on several phenols not thought to be associated with polycarbonate bottle use. As expected, we observed no difference for MePB, PrPB, or TCS, although urinary concentrations of BP-3 were higher after polycarbonate bottle use. However, after stratification by percent compliance during the intervention week, the association for BP-3 was stronger in the less compliant group. By contrast, the association between BPA and poly-carbonate bottle use was stronger in the more compliant group, suggesting that BPA may leach from polycarbonate bottles. We found BPA and BP-3 to be strongly correlated: The Pearson correlation coefficients between BP-3 and BPA were 0.38 (*p* = 0.0008) and 0.43 (*p* = 0.0001) during the washout week and intervention week, respectively. Although this study was not designed to look at other sources of BPA, or any sources of the other phenols, we hypothesize that the strong correlation observed between BPA and BP-3 could be the result of a shared source or behavior. We are not aware of the presence of BP-3—a common sunscreen agent in personal care products—in polycarbonate plastic, although it can also be used as ultraviolet stabilizer in plastic surface coatings for food packaging to prevent polymer or food photo degradation (Suzuki et al. 2005). However, because sources and routes of exposure for many of these compounds are not yet known, it is possible that BPA and BP-3 are used in a common product that has not yet been identified. An alternative explanation is that students who participated in the most outdoor physical activity drank the most fluid from their bottles and also applied the most sunscreen, potentially increasing both BPA and BP-3 levels.

Our study population included a high proportion of Asian and Caucasian participants, and our participants were young. However, there is no obvious reason why the results of our study should not apply to other ethnicities and age groups. Furthermore, the use of polycarbonate bottles is very popular among college students, making this an especially relevant population to study. Although we assessed the effect of the exclusive use of poly-carbonate plastic bottles as beverage containers, a proportionate increase in urinary BPA would be expected among individuals who use polycarbonate plastic bottles in combination with other beverage containers. Children have been found to have higher urinary BPA concentrations than adolescents and adults (Calafat et al. 2008), consistent with animal evidence of reduced glucuronidation in fetuses and neonates (Matsumoto et al. 2002). Thus, because of their reduced ability to clear BPA, we predict that children would have higher urinary BPA concentrations due to use of polycarbonate plastic bottles relative to the study population.

The major strength of this study is the non-randomized intervention design. We compared urinary BPA concentrations within each participant, which precluded confounding by subject characteristics that remain constant over time. Although within-person confounding was possible, it is unlikely that unmeasured confounding could account for the large effect estimate we observed. The large increase in mean urinary BPA concentration after regular use of polycarbonate bottles suggests that the systematic BPA variation in the two study phases was by far greater than any random variation due to BPA ingestion from other sources.

Furthermore, we assessed the impact of polycarbonate bottle use in a normal use setting. The present study could be considered a conservative estimate of true use, as students did not have access to dishwashers and were instructed to use their containers for cold beverages only, whereas the storage of hot liquids is common, especially in outdoor recreation settings. Because heating is thought to increase the amount of BPA leached from the polycarbonate (Le et al. 2008), we would anticipate higher urinary BPA concentrations after ingestion of hot beverages stored in the same bottles.

## Conclusions

To our knowledge, this is the first study to assess the impact of polycarbonate drinking bottle use on urinary BPA concentrations. Despite within-person variability resulting from other sources of BPA exposure, a measurable increase in urinary BPA resulted from only 1 week of exposure to beverages contained in polycarbonate bottles. Replication of this study in other populations may help to inform public health policy regarding the use of BPA in polycarbonate food and beverage containers.

## Figures and Tables

**Table 1:** Characteristics of 77 Harvard College students enrolled in a nonrandomized intervention study assessing changes in urinary phenol concentrations associated with use of polycarbonate drinking containers.

Characteristic	No. (%)
Sex	
Male	41 (53.2)
Female	36 (46.8)
Ethnicity	
Caucasian	30 (39.0)
Asian	38 (49.3)
African American	5 (6.5)
Hispanic	4 (5.2)
Percent compliance [median of proportion (range)]	90 (50–100)
Age, years [median (range)]	19 (18–23)

**Table 2:** GM concentrations of phenols (μg/creatinine) after washout and intervention.

Phenol	Week of study	GM (95% CI)
BPA	Washout	1.2 (1.0–1.4)
	Intervention	2.0 (1.7–2.4)
BP-3	Washout	46.1 (30.6–69.5)
	Intervention	66.8 (42.3–105.5)
MePB	Washout	51.3 (37.3–70.7)
	Intervention	48.4 (36.2–64.8)
PrPB	Washout	8.4 (5.4–12.9)
	Intervention	8.8 (5.8–13.1)
TCS	Washout	15.5 (9.5–25.3)
	Intervention	17.3 (10.7–28.1)

Concentrations (μg/L) < LOD were recorded as 1/2 LOD, which is 0.2 for BPA and BP-3; 1.15 for TCS; 0.5 for MePB; and 0.1 for PrPB.

**Table 3:** Percent change in urinary concentrations of phenols associated with 1-week use of polycarbonate drinking containers.

Phenol	Percent change (95% CI)	*p*-Value	*p* for heterogeneity
BPA			
Overall	69 (40 to 102)	< 0.0001	
≥ 90% compliance	77 (45 to 117)	< 0.0001	
< 90% compliance	55 (6 to 127)	0.03	0.54

BP-3			
Overall	45 (16 to 81)	0.001	
≥ 90% compliance	36 (2 to 80)	0.04	
< 90% compliance	64 (11 to 142)	0.01	0.42

MePB			
Overall	−6 (−25 to 18)	0.60	
≥ 90% compliance	17 (−10 to 51)	0.24	
< 90% compliance	−34 (−56 to 0)	0.05	0.01

PrPB			
Overall	5 (−24 to 44)	0.77	
≥ 90% compliance	15 (−23 to 70)	0.49	
< 90% compliance	−10 (−49 to 59)	0.70	0.46

TCS			
Overall	12 (−17 to 50)	0.46	
≥ 90% compliance	11 (−18 to 50)	0.50	
< 90% compliance	17 (−39 to 126)	0.62	0.88

Concentrations (μg/L) < LOD were recorded as 1/2 LOD, which is 0.2 for BPA and BP-3; 1.15 for TCS; 0.5 for MePB; and 0.1 for PrPB. Twenty-eight participants reported < 90% compliance over intervention week, 48 participants reported ≥ 90% compliance, and compliance was missing for one participant.
